# Programming experience associated with neural efficiency during figural reasoning

**DOI:** 10.1038/s41598-020-70360-z

**Published:** 2020-08-07

**Authors:** Birgit Helmlinger, Markus Sommer, Martina Feldhammer-Kahr, Guilherme Wood, Martin E. Arendasy, Silvia E. Kober

**Affiliations:** 1 Institute of Psychology, University of Graz, Universitätsplatz 2, 8010 Graz, Austria; 2 BioTechMed-Graz, Graz, Austria

**Keywords:** Psychology, Cognitive neuroscience, Computational neuroscience

## Abstract

In the present study, we investigated neural processes underlying programming experience. Individuals with high programming experience might develop a form of computational thinking, which they can apply on complex problem-solving tasks such as reasoning tests. Therefore, *N* = 20 healthy young participants with previous programming experience and *N* = 21 participants without any programming experience performed three reasoning tests: Figural Inductive Reasoning (FIR), Numerical Inductive Reasoning (NIR), Verbal Deductive Reasoning (VDR). Using multi-channel EEG measurements, task-related changes in alpha and theta power as well as brain connectivity were investigated. Group differences were only observed in the FIR task. Programmers showed an improved performance in the FIR task as compared to non-programmers. Additionally, programmers exhibited a more efficient neural processing when solving FIR tasks, as indicated by lower brain activation and brain connectivity especially in easy tasks. Hence, behavioral and neural measures differed between groups only in tasks that are similar to mental processes required during programming, such as pattern recognition and algorithmic thinking by applying complex rules (FIR), rather than in tasks that require more the application of mathematical operations (NIR) or verbal tasks (VDR). Our results provide new evidence for neural efficiency in individuals with higher programming experience in problem-solving tasks.

## Introduction

There is a general agreement that computational thinking (CT) is one of the most essential skills in the context of the twenty-first century’s steadily progressing digitalization. This postulation originates from a viewpoint article published by Wing in 2006, where she postulated that CT, alongside reading, writing, and arithmetic, is a fundamental skill that everybody should learn, not only computer scientists (^1^, p. 33). Wing’s article drew general attention to CT, triggering a huge wave of research, especially in the field of education^2,3^. Additionally, a series of training programs were developed to help children^4^ as well as adults^5,6^ to acquire higher levels of CT.

Although the number of basic research and training studies on the topic of CT is rising, there is still no consensus about its definition^3,7,8^. In a recent review article, Shute et al.^3^ tried to find the similarities between the several definitions and defined CT as “*the conceptual foundation required to solve problems effectively and efficiently (i.e., algorithmically, with or without the assistance of computers) with solutions that are reusable in different contexts*” (^3^, p. 142). Hence, CT is associated with a set of skills including algorithmic and logical thinking, problem-solving as well as efficient and innovative thinking^3,9^. These mental processes are also involved in programming. Hence, CT and programming skills are strongly interrelated but not equivalent^3,10,11^. It is assumed that successful programming and coding requires CT skills (e.g., abstraction, decomposition, algorithmic thinking, debugging, iteration, and generalization), “*but considering CT as knowing how to program may be too limiting*” (^3^, p. 142). Most researchers agree with this assumption that the two constructs are related, but not identical^12,13^. However, since CT is required in programming^1,3,10,11,14^, CT assessment is often based on programming environments^15^. Similarly, programming interventions have been used to increase CT skills in many studies^3,16^.

Thus, since CT skills are required for programming^1,3,10,14^, we assume that individuals with high programming experience develop a form of CT, which they can apply on complex problem-solving tasks that go beyond mere programming, such as reasoning tests. Therefore, we decided to investigate two groups, one with no programming experience and one group with prior programming experience, while performing tasks that require problem-solving, algorithmic and logical thinking as well as efficient and innovative thinking. The tasks we used here were part of a fluid intelligence test. Fluid intelligence (G_f_ = reasoning) is, besides crystallized intelligence (G_c_), one of the two facets of general intelligence (G). While fluid intelligence refers to the ability to solve novel reasoning problems, which requires skills such as comprehension, problem-solving, and learning, crystallized intelligence refers to knowledge that comes from prior learning and past experiences^17^. Fluid intelligence was assessed using three subtests of the Intelligence-Structure-Battery 2 (INSBAT 2:^18^), namely, figural inductive reasoning (FIR), numerical inductive reasoning (NIR), and verbal deductive reasoning (VDR). For a comprehensive description of these tasks please see the methods section. Empirical studies indicated that higher CT and programming skills come along with higher reasoning skills^14,19–21^. Training of CT skills as well as programming skills has been shown to lead to an improvement in figural reasoning tasks but not in numerical or verbal reasoning tasks^13,14,20,22,23^. Figural reasoning skills even turned out to be one of the best predictors of learning outcomes when learning a programming language such as Python, while numeracy only explained a relatively small portion of variance in programming learning outcomes^21^. This indicates that figural reasoning is particularly relevant for programming and might also play a central role in CT.

Besides differences in performance between programmers and non-programmers in complex problem-solving tasks, such as represented in reasoning tests, we are further interested in differences in neural processes underlying programming experience when performing such tasks. From a neuroscientific viewpoint, there are only a few studies that investigated neural correlates of programming experience or CT, respectively. Using EEG measurements, Park, Song and Kim (2015) investigated the relation between cognitive load related to programming experience and CT^24^. Generally, an increase in cognitive load while performing cognitive tasks is associated with changes in two distinct EEG frequency bands: a task-related decrease in alpha (8–12 Hz) power (event-related desynchronization, ERD) and a task-related increase in theta (4–8 Hz) power (event-related synchronization, ERS)^25–29^. The aim of the study by Park et al.^24^ was to compare the effects of two different programming courses (programming courses based on Scratch vs. programming courses based on Scratch + additional CT teaching) on university students’ problem-solving ability and cognitive load while working on problem-solving tasks. The group with additional CT teaching showed higher improvement in CT-based problem-solving tasks than the other group. As for the EEG assessment, no significant differences in cognitive load were observed between groups. However, EEG was only recorded over two frontopolar electrode positions, limiting the significance of the EEG results. Although no group differences in cognitive load were observed according to the EEG results, the authors reported that the group with additional CT teaching tended to approach the problems more efficiently, as indicated for instance by improved strategic thinking, simultaneous thinking, and the use of recursive solution strategies during the problem-solving processes^24^. In line with that, there is strong evidence that people with higher cognitive abilities (e.g., individuals with higher intelligence) show more efficient, thus, lower cortical activation when performing cognitively demanding tasks (such as reasoning tasks) than people with lower cognitive abilities. Furthermore, it is suggested that neural efficiency does not only indicate lower cortical activation, but also more locally focused activation in task-relevant brain areas^30–34^. Concerning brain connectivity measures, prior studies report conflicting results that either increased or reduced brain connectivity each might be a sign of neural efficiency^35–37^. In summary, higher programming skills might lead to a more efficient neural processing when performing reasoning tasks.

A more efficient neural processing in programmers than in non-programmers might be related to a stronger automation of critical skills needed to solve such complex reasoning tasks. According to the dual-process theory, mental activity involved in performing reasoning and decision making tasks, for instance, is categorized in two main types of processing: type I processes including more automatic and capacity-free processes (fast, high capacity, independent of working memory) and type II processes including more controlled and capacity-limited processes (slow, low capacity, heavily dependent on working memory)^38,39^. Note that type I and type II processes are highly interdependent. Type I and type II processes are associated with activation in distinct brain networks. Type II processes are linked to frontal executive functions (top-down control) while type I processes are thought to result from relative hypofrontality^40–42^. Type II processes reflect the activity of a supervisory attention system, specialized in monitoring and regulating the activity in other cognitive/neural systems^43^. Hence, differences in brain activation and connectivity between programmers and non-programmers when solving reasoning tasks might be caused by a stronger involvement of type I processes in programmers and a stronger involvement of type II processes in non-programmers.

In the present study, we compare individuals with and without previous programming experience while solving figural, numerical, and verbal reasoning tasks with different levels of complexity (three levels of difficulty) in (1) behavioral performance and (2) neural processing. We expect that programmers, who might have developed a form of CT, which is required to program successfully^1,3,10,11,14^, show a better performance in the reasoning tasks than non-programmers. This group difference in behavioral performance should be larger in tasks requiring figural reasoning^13,14,20,22,23^.

Additionally, we expect that group differences in behavior go along with group differences in neural correlates underlying cognitive processing. In accordance with the neural efficiency theory as well as the dual-process theory, we hypothesize that programmers, who should show a superior performance especially in figural reasoning, display more efficient neural processing probably due to a more effortless and automatic task processing (type I processes) as compared to non-programmers^30–34^. A more efficient neural processing should be seen in a less pronounced alpha ERD^33,44^ and a less pronounced theta ERS^45^. Concerning brain connectivity, we also expect differences between groups while solving reasoning tasks ^35–37^. Since we assume that non-programmers show a stronger involvement of type II processes when solving reasoning tasks, it might be that this group shows a stronger connectivity between frontal brain areas and more parietal brain areas due to a stronger executive control^41,46^.

Exploratively, we assess mental strategies used by participants to solve the reasoning tasks. Verbal reports may provide insight into various strategies for solving problems and might be related to differences in brain activity^47^.

## Methods

### Participants

In the present study, we compared two groups of university students, namely students with and without prior programming experience. To assess the level of programming experience prior to the EEG measurement and to find two homogenous groups (comparable in age and gender), 273 potential participants filled out a short electronic questionnaire (22 questions). In this questionnaire, we asked for information about programming experiences within the school education, the study career, further education, during their leisure time, or any other possible occupation. The last question (“expertise-rating”) asked participants to self-rate their current programming knowledge on a visual analogue scale from layman (= 0) to expert (= 10). To be eligible for the programming group (“programmers”), participants had to state a value of 5 or higher in the expertise-rating. If a value of 0 was entered, and participants did not state to have obtained programming experience in any of the other questions, participants were considered for the non-programmers’ group (“non-programmers”; descriptive statistics of the expertise-rating in programmers’ group: *M*_male_ = 6.92, *SD*_male_ = 1.51; *M*_female_ = 6.25, *SD*_female_ = 0.89). There is evidence that programming experience can be reliably assessed using such self-estimation ratings^48^. Finally, two exclusion criteria were applied for all participants: i) skin intolerances of the electrode paste; and ii) neurological diseases.

The final sample that completed the EEG measurement consisted of 41 university students (22 men, 19 women) between 20 and 39 years (*M* = 24.95 years, *SD* = 3.94). Twenty participants were in the programmers’ group (12 men, 8 women, mean age = 25.40 years, *SD* = 3.98), twenty-one participants were non-programmers (10 men, 11 women, mean age = 24.52 years, *SD* = 3.96). Table A1 of the Supplementary Material summarizes prior programming experience and education of both groups in more detail. All volunteers gave their written informed consent. The study was approved by the local ethics committee of the University of Graz, Austria (GZ. 39/11/63 ex 2018/19) and is in accordance with The Code of Ethics of the World Medical Association (Declaration of Helsinki) for experiments involving humans^49^. Volunteers were paid for their participation (24€).

### Assessment of reasoning

Reasoning (G_f_: fluid intelligence) was measured by means of three subtests taken from the Intelligence-Structure-Battery 2 (INSBAT 2:^18^). This intelligence test battery is widely used in German-speaking countries and is based on the Cattell–Horn–Carroll model (CHC-model:^50,51^). INSBAT 2 assesses the second stratum factors fluid intelligence (G_f_), crystallized intelligence (G_c_), quantitative knowledge (G_q_), visual processing (G_v_), and long-term memory (G_lr_) by means of two to three subtests. All subtests were constructed using automatic item generation (AIG:^52,53^) on the basis of a cognitive processing model, which outlines the cognitive processes test-takers use to solve these tasks in addition to the item design features linked to these cognitive processes. All subtests were calibrated by means of the 1PL Rasch model^54^ and have been shown to exhibit good construct and criterion validities (for an overview:^18^). In the present study only the three subtests (FIR: figural inductive reasoning, NIR: numerical inductive reasoning, VDR: verbal deductive reasoning) measuring fluid intelligence (G_f_) were used. These three subtests were chosen based on factor analytic evidence indicating that individual differences in commonly used fluid intelligence tasks are best modeled by a general fluid intelligence factor and modality-specific factors (e.g., reflecting figural reasoning; cf.^55–57^).

**Figure 1:**
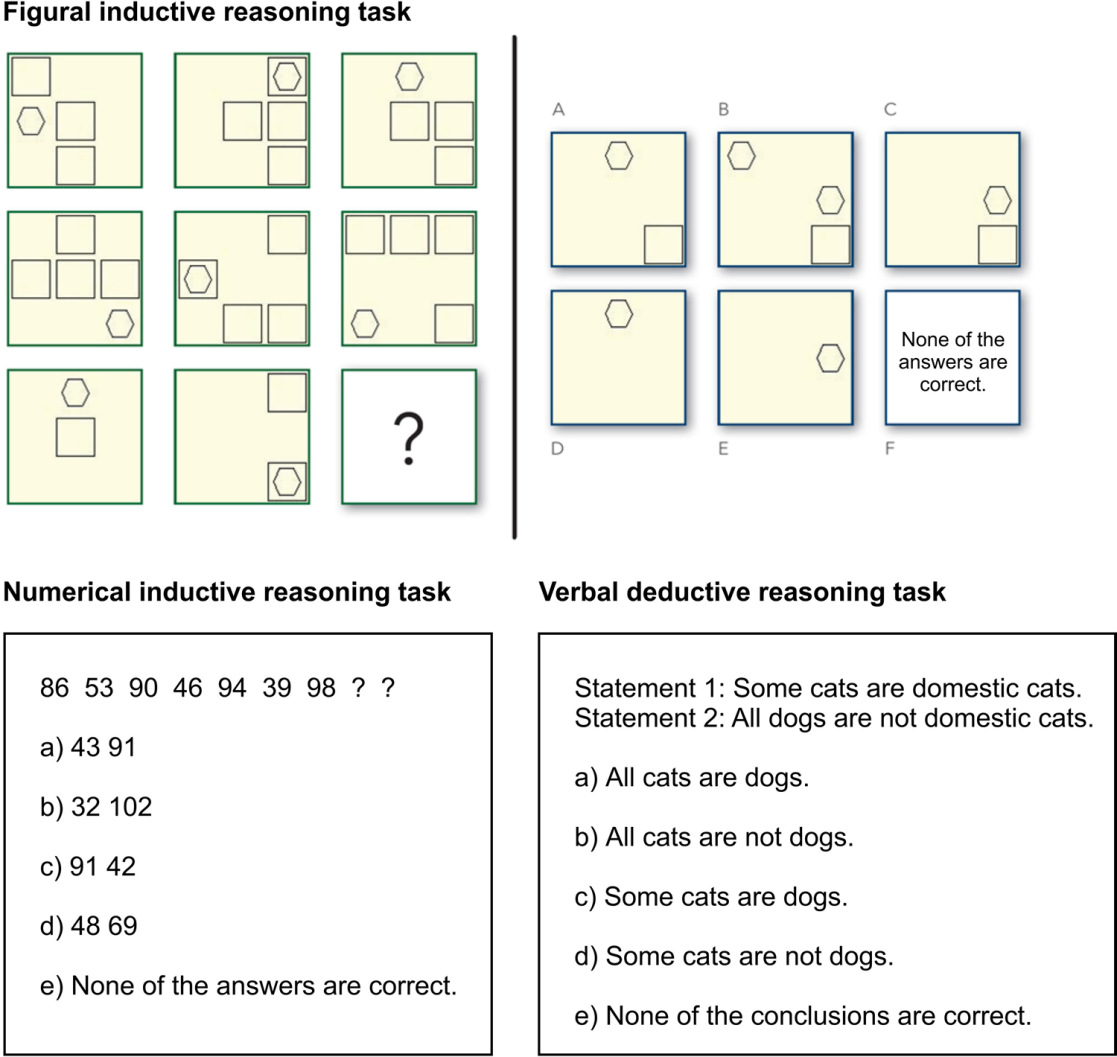
Example items of the three reasoning tasks of the Intelligence-Structure-Battery 2 (INSBAT 2:^18^). All three items are examples for medium complex items. In the Figural Inductive Reasoning (FIR) task, the left side shows a 3 × 3-matrix with one missing field. The right side shows 6 possible response options (correct answer for this example item: C). In the Numerical Inductive Reasoning (NIR) task, a sequence of numbers that has to be continued is shown in the first row and the five response options are underneath (correct answer for this example item: B). In the Verbal Deductive Reasoning (VDR) task, the two statements are presented on top, the four possible conclusions and the fifth response option are shown underneath (correct answer for this example item: D).

In INSBAT, all subtests are commonly administered as computerized adaptive tests (CAT:^58^) with a target reliability corresponding to Cronbach’s *α* = 0.70. Due to our EEG paradigm, however, it was more appropriate to administer these three subtests as fixed-item linear tests. Furthermore, the present research design required the use of an approximately equal number of items that exhibit low, medium, and high difficulties (i.e. levels of complexity). To achieve these two aims, a total of k = 7 items of low, medium, and high difficulty (three complexity levels) were randomly drawn from the current item pool. This yielded a total of k = 21 items for each of the three subtests. The same 21 items were presented in the same order for all participants per task (FIR, NIR, VDR). All three tests were computerized using the program PsychoPy^59^. For each of the three subtests, participants had a maximum of 30 min to complete the fixed-item linear test forms. As soon as an answer was given, a fixation cross was displayed in the center of the screen for nine seconds, followed by the next item. Participants were instructed that the test would be terminated if duration exceeded 30 min. For one participant (non-programmer), the FIR was stopped manually because the time was exceeded. In Fig. 1 example items for each task are illustrated.

#### Figural inductive reasoning (FIR)

In this subtest, participants had to infer the rules governing figural matrices and to complete the matrices by applying these rules. They were presented with k = 21 3 × 3-matrices. The first eight cells of each matrices were filled with geometrical figures (e.g. rectangles, circles, etc.) while the bottom right field was always empty. The number and arrangement of the geometrical figures followed certain rules that had to be inferred to solve the test item (for further details:^60–62^). Respondents were presented with six response options, including the response option “none of the answer alternatives is correct.”. This response option was included to prevent respondents from resorting to response elimination to solve the test items^62^. They were asked to press one of six keys on a conventional keyboard to indicate which answer alternative they considered to be the correct solution. The test items were constructed by means of AIG on the basis of cognitive processing models for figural matrices (e.g.^63,64^). Prior research indicated that these items measure fluid intelligence and exhibit a g-factor saturation comparable to commonly used figural matrices tests such as the Ravens matrices (cf.^18,60,61,65^). Furthermore, item design features linked to cognitive processes involved in solving figural matrices tests have been shown to account for 91.8% of the differences in the 1PL item difficulty parameters^18,62^. Thus, there is evidence on the construct validity of the figural matrices items used in the present study.

#### Numerical inductive reasoning (NIR)

In this subtest, the task of the participants was to discover the rules that govern a number series, and to continue the number series by applying these rules. They were administered k = 21 number series consisting of seven numbers each, constructed under certain rules, and four response alternatives in addition to the response alternative “none of the answer alternatives is correct.” to prevent response elimination (for further details:^52^). Similar to the figural matrices test the items of this subtest were constructed on the basis of cognitive processing models for number series tasks (e.g.^66,67^) using AIG. Prior research indicated that the number series task used in this study measures fluid intelligence and exhibit a g-factor saturation comparable to the Ravens matrices (cf.^18,52,65^). In addition, item design features linked to cognitive processes hypothesized to be involved in solving number series have been shown to account for 88.2% of the differences in the 1PL item difficulty parameters^18,52^. Taken together these results argue for the construct validity of the number series used in the present study.

#### Verbal deductive reasoning (VDR)

This subtest consisted of k = 21 syllogism tasks. Each test item consisted of two statements (premises) and four possible conclusions in addition to the response alternative “none of the conclusions is logically valid.”. The participants were instructed to assume that the premises were true, and to indicate, which of the four possible conclusions—if any—follows logically from the given premises. As outlined by Arendasy, Sommer, and Gittler^18^ the items were constructed by means of AIG on the basis of current cognitive processing models for syllogistic reasoning tasks (e.g.^68–70^) by systematically manipulating the item design features figure of the syllogism, cognitive complexity of the premises, plausibility, and falsification difficulty. Prior research indicated that these item design features and the cognitive processes linked to them accounted for 83.2% of the differences in the 1PL item difficulty parameters^18^. Furthermore, factor analytic research indicated that this subtest measures fluid intelligence and exhibits a high g-factor saturation, which argues for the construct validity of this measure (cf.^18,52,65^).

After completing each of the three reasoning tasks, participants were asked to report the strategies they used to solve the items by filling in a blank box on a sheet of paper. They were free to decide whether they wanted to write down whole sentences or just some keywords. Participants were allowed to describe as many strategies as they wanted.

### EEG recording and data analysis

EEG was recorded with 60 active electrodes (placed in accordance with the 10–20 EEG placement system^71^) using two BrainAmp 32 AC EEG amplifiers from Brain Products GmbH (Gilching, Germany). The ground was placed at Fpz, the linked references were placed on the left and right mastoid. Ocular artifacts were recorded with three EOG electrodes placed at the left and right temples and the nasion. The impedances of all EEG and EOG electrodes were kept below 25 kΩ. The sampling rate was 500 Hz. We used a 70 Hz low pass filter, a 0.01 Hz high pass filter, and a 50 Hz Notch filter.

Before the start of the INSBAT tasks, resting measurements with open and closed eyes were performed (one minute each). Analysis and results of these resting measurements can be found in Supplementary Material B.

For EEG data analysis, we used the Brain Vision Analyzer (version 2.01, Brain Products GmbH, Gilching, Germany). First, the raw data were inspected visually to remove major muscle artifacts. Following this, eye-movement artifacts were removed semi-automatically by Independent-Component-Analysis (ICA, Infomax). Additionally, a semi-automatic artifact correction was performed with the following criteria: within a 100 ms interval, only voltage fluctuations between 0.5 and 50 µV and amplitudes between −150 and 150 µV were allowed^72,73^. All epochs with artifacts were excluded from the EEG analysis.

Alpha- (8–12 Hz) and theta band power (4–8 Hz) were extracted by means of the Brain Vision Analyzer’s built-in function of complex demodulation^72,74^. To analyze task-related power changes in the reasoning tasks, we calculated the percentage power changes from a baseline (i.e. the time before stimulus-onset) to an active phase (i.e. the time during which the stimulus was processed). This is represented by the following equation ((active phase – baseline)/baseline*100)^29^. Therefore, decreases in power compared to the baseline result in negative values, representing event-related desynchronization (ERD), and increases in power in positive values, representing event-related synchronization (ERS). An ERD is caused by a decrease and an ERS is caused by an increase in synchrony of the underlying neuronal populations, respectively^29^. An ERD in the alpha frequency range (relative task-related power decrease from baseline to an active phase, suppression of alpha oscillations) is associated with neural activation since alpha oscillations are related to an inactive resting state as well as active inhibition of brain areas where alpha oscillations are pronounced strongly^29,75^. The alpha rhythm is predominant in healthy humans and most pronounced over posterior regions (e.g., parietal, occipital) of the brain^25,28^. An alpha ERD can be seen while performing a variety of tasks such as perceptual, judgement, memory, or motor tasks. Generally, an increase of task complexity or attention results in an increased magnitude of alpha ERD (for an overview see^29^). In contrast, a task-related increase in theta power (theta ERS) is generally associated with encoding of new information, episodic memory, and working memory^28^. Theta is mainly seen in the frontal midline area^25,28^. In the present study, we especially focused on alpha and theta frequencies since different studies showed that changes in these two EEG frequencies are reliable indicators for changes in task difficulty or cognitive load in a variety of task demands^25,27,28^.

For calculating ERD/ERS values in the present study, the active phase was defined as the time between stimulus onset (first presentation of an item on the screen) and response of the participant (pressing a response key). The baseline interval for each item was 6,000 ms before stimulus onset to stimulus onset. Both baseline and active phase were again split into segments of two seconds and each segment that contained an artifact was excluded from further processing. The power in all remaining 2-s segments was averaged per EEG channel. Note that only correctly answered items were analyzed. ERD/S values were averaged separately for each reasoning task (FIR, NIR, VDR) and complexity level (low, medium, high). Additionally, single electrode positions were merged to regions of interest (ROIs). For alpha ERD/ERS, ten parieto-occipital electrodes (5 each) were merged to two ROIs: left parietal (P1, P3, P5, PO3, PO7) and right parietal (P2, P4, P6, P04, P08) ROI. For theta ERD/ERS, electrodes AFz, Fz, and FCz were merged to one fronto-central ROI.

To analyze EEG coherence, the active phase of correctly answered items of the reasoning tasks was cut in artifact-free 2-s epochs. A FFT transformation was performed per epoch (Hanning window, 10%). Then, the magnitude-squared coherence was calculated for the channel pairs connecting fronto-parietal areas (left: AF3, F3, FC3 with PO3, O1; middle: AFz, Fz, FCz with POz, Oz; right: AF4, F4, FC4 with PO4, O2) and average coherence values in the frequency range of 4–8 Hz and 8–12 Hz were extracted per reasoning task (FIR, NIR, VDR) and complexity level (low, medium, high). Coherence is a frequency domain measure of the functional coupling or similarity between signals assessed at two different electrode positions. The magnitude-squared coherence estimates the linear relationship of two signals at each frequency bin on the basis of cross- and auto-spectra of the involved signals^72^. Values can range from 0 (no similarity/functional coupling between signals assessed at two different brain areas) to 1 (maximum similarity/functional coupling between signals assessed at two different brain areas).

### Statistical analysis

To analyze possible group differences in the performance of the three reasoning tasks, the number of correctly answered items in each of the three tests (FIR, NIR, VDR) was compared using analyses of covariance (ANCOVAs). These ANCOVAs were performed with Group (programmers, non-programmers) as a between-subjects factor and Complexity (low, medium, high) as a within-subjects factor. Age and sex of the participants were used as covariates in the analyses because sex and age might have an influence on brain activation as well as performance in reasoning or working memory tasks^28,76,77^. Reaction times were not investigated in the present study, because participants were not asked to answer as quickly as possible, but only to try to solve all items of a task within 30 min.

The subjectively reported mental strategies, displaying how the participants have solved the reasoning tasks, were divided into different categories (Table 2)^78^. Note that each participant could report more than only one strategy per task. Absolute frequencies of the reported mental strategies were statistically compared between tasks (FIR, NIR, VDR) as well as between groups within each task using χ^2^ tests.

To analyze possible group differences in ERD/S values, several ANCOVAs for each of the three reasoning tests (FIR, NIR, VDR) were carried out. ANCOVAs were conducted separately for alpha ERD/S and frontal theta ERD/S as dependent variables. Similar to the behavioral analyses, the between-subjects-factor Group (programmers, non-programmers) and the within-subjects factor Complexity (low, medium, high) were included in all ANCOVAs. For frontal theta ERD/S, no additional within-subjects factor was used. For alpha ERD/S, another within-subjects-factor concerning the ROIs (left, right) was added. Age and sex of the participants were used as covariates.

To analyze possible group differences in coherence values, several ANCOVAs for each of the three reasoning tests (FIR, NIR, VDR) were carried out. ANCOVAs were conducted separately for coherence in the alpha and theta frequency range as dependent variables. The ANCOVA models comprised the between-subjects-factor Group (programmers, non-programmers) and the within-subjects factors Complexity (low, medium, high) and Hemisphere (left, middle, and right fronto-parietal connections). Age and sex of the participants were used as covariates.

For all analyses, degrees of freedom for each analysis were adjusted using the Greenhouse–Geisser procedure to correct for violations in sphericity if necessary. Significance level was set at 0.05, except for multiple t-tests (e.g. differences in possible confounders and post-hoc tests). Adjustment for multiple comparisons was done with Holm–Bonferroni method.

## Results

### Behavioral results

For the FIR task, the ANCOVA revealed a significant main effect of Group (*F*(1,36) = 16.22, *p* < 0.0001, *η*_*p*_^*2*^ = 0.31) with programmers showing generally more correctly answered items than non-programmers. Additionally, a significant Complexity*Group interaction was found (*F*(2,72) = 7.01, *p* < 0.01, *η*_*p*_^*2*^ = 0.16). Post-hoc comparisons revealed that programmers performed significantly better than non-programmers in the medium (*p* < 0.001) and highly complex tasks (*p* = 0.004), but not in the low complex tasks (*p* = 0.537). The covariates sex and age had no significant effects. Means and *SE* of all behavioral results are shown in Table 1.

The ANCOVA for the number of correctly answered NIR items revealed a significant main effect of Complexity (*F*(1.65,61.20) = 6.38, *p* < 0.01, *η*_*p*_^*2*^ = 0.15). Post-hoc comparisons showed that all participants, regardless of their group membership, correctly answered more low than medium and highly complex items, and more medium than highly complex items (all *p* < 0.001, Table 1). The covariates were non-significant. The ANCOVA for the VDR task revealed no significant results (Table 1).

**Table 1:** Means (*M*) and standard errors (*SE*) for the number of correct responses per group (programmers, non-programmers), reasoning task (FIR, NIR, VDR), and complexity level (low, medium, high).

	Programmers			Non-programmers		
	*N*	*M*	*SE*	*N*	*M*	*SE*
*FIR*						
Low	20	5.65^c, d^	0.18	20	5.40 ^e, f^	0.23
Medium	20	4.80^a, c^	0.28	20	2.95^a, e^	0.31
High	20	4.10^b, d^	0.36	20	2.60^b, f^	0.38
*NIR*						
Low	20	6.20	0.25	21	6.00	0.23
Medium	20	4.95	0.27	21	4.24	0.41
High	20	3.10	0.27	21	2.48	0.35
*VDR*						
Low	20	5.15	0.18	21	4.76	0.22
Medium	20	5.75	0.22	21	5.48	0.25
High	20	4.30	0.36	21	3.52	0.34

*FIR* figural inductive reasoning, *NIR* numerical inductive reasoning, *VDR* verbal deductive reasoning. *low* low complexity, *medium* medium complexity, *high* high complexity. Superscripted letters indicate significant differences revealed by the post-hoc tests for the interaction effect Complexity*Group.

### Mental strategies used to solve the reasoning tasks

Table 2 summarizes the relative frequencies of mental strategies reported per group when solving the three reasoning tasks (FIR, NIR, VDR) per group (programmers, non-programmers) and the results of the statistical comparisons. There were no large differences in the mental strategy report between programmers and non-programmers (Table 2). Programmers only reported the use of the strategy “*Finding differences in response options*” during the FIR task (*χ*^*2*^(1) = 4.65, *p* < 0.05, *Cramer’s V* = 0.337) and “*Rejecting wrong answers step by step*” during the NIR task (*χ*^*2*^(1) = 4.65, *p* < 0.05, *Cramer’s V* = 0.337) more often than non-programmers. Hence, programmers and non-programmers reported the use of the single mental strategies per task with a largely comparable frequency. Therefore, absolute frequencies of the reported mental strategies were statistically compared between the three tasks for the merged data of programmers and non-programmers. During the FIR task, participants reported to use many different strategies focusing on the elements of the items (number, position, shape, rotation of objects). Systematically analyzing the rows and columns of the items was also only reported for the FIR task. Pattern recognition was also more frequently reported after the FIR task than after the NIR and VDR task. In contrast, the use of numerical operations was most frequently reported for the NIR task. In this task, analyzing the characteristics of neighboring numbers was reported, too. Detecting rules or similarities was equally often used for the FIR and NIR task, but this strategy was not used for the VDR task. Abstract thinking, deductive reasoning, and visual imagery of solutions was only reported for the VDR task. The reported solution strategies are in line with the cognitive processes hypothesized to be involved in solving these tasks (cf. methods section). Furthermore, the results are also consistent with prior studies indicating that item design features linked to these cognitive processes account for 83.2% to 91.8% of the differences in the 1PL item difficulty parameters of the three reasoning tests (for an overview:^18^).

**Table 2:** Relative frequencies of reported mental strategies (per group and task) used to solve the three reasoning tasks (FIR, NIR, VDR) per group (programmers, non-programmers) and results of statistical comparisons of absolute frequencies of the reported mental strategies between the three tasks (merged for programmers and non-programmers).

Mental strategy	FIR		NIR		VDR		*Χ*^*2*^	df	*P*	*Cramer’s-V*
	Pr	N-Pr	Pr	N-Pr	Pr	N-Pr				
Number of objects	0.30	0.48	0.00	0.00	0.00	0.00	36.79	2	0.000	0.547
Position of objects	0.40	0.52	0.00	0.00	0.00	0.00	44.94	2	0.000	0.604
Shape of objects	0.55	0.71	0.00	0.00	0.00	0.00	65.94	2	0.000	0.732
Rotation of objects	0.20	0.14	0.00	0.00	0.00	0.00	14.85	2	0.001	0.347
If–then/or operations	0.05	0.00	0.00	0.00	0.00	0.00	2.02	2	0.365	0.128
Pattern recognition	0.75	0.67	0.50	0.33	0.15	0.00	34.46	2	0.000	0.529
Numerical operations + /-/*/ ÷ / Magnitudes	0.10	0.10	0.80	0.81	0.20	0.10	56.28	2	0.000	0.676
Finding differences in response options	0.20^a^	0.00^a^	0.00	0.00	0.00	0.00	8.27	2	0.016	0.259
Logical thinking	0.05	0.14	0.10	0.14	0.20	0.29	3.86	2	0.145	0.177
Analyzing rows and columns	0.40	0.38	0.00	0.00	0.00	0.00	36.79	2	0.000	0.547
Rejecting wrong answers step by step	0.40	0.19	0.20^b^	0.00^b^	0.40	0.19	5.92	2	0.052	0.219
Guessing	0.15	0.19	0.15	0.19	0.10	0.05	2.18	2	0.336	0.133
Detecting rules/similarities	0.30	0.29	0.40	0.43	0.00	0.00	20.67	2	0.000	0.410
Characteristics of neighboring numbers	0.00	0.00	0.25	0.52	0.00	0.00	36.79	2	0.000	0.547
Abstract thinking	0.00	0.00	0.00	0.00	0.10	0.05	6.15	2	0.046	0.224
Deductive reasoning	0.00	0.00	0.00	0.00	0.30	0.33	29.07	2	0.000	0.486
Visual imagery of premises/solutions	0.00	0.00	0.00	0.00	0.20	0.24	19.42	2	0.000	0.397
Going through premises backwards	0.00	0.00	0.00	0.00	0.10	0.00	4.07	2	0.131	0.182
Tautology	0.00	0.00	0.00	0.00	0.05	0.00	2.02	2	0.365	0.128
Attention to words "none", "no one", "all", "some", etc	0.00	0.00	0.00	0.00	0.05	0.05	4.07	2	0.131	0.182
No strategy	0.00	0.00	0.00	0.00	0.00	0.05	2.02	2	0.365	0.128

*FIR* figural inductive reasoning, *NIR* numerical inductive reasoning, *VDR* verbal deductive reasoning. *Pr* Programmer, *N-Pr* non-programmer. Superscripted letters indicate significant differences between groups per task.

### Task-specific EEG power changes

Table 3 summarizes the alpha ERD/S values for each reasoning task and each complexity level per group.

**Table 3:** Means (*M*) and standard errors (*SE*) for parieto-occipital alpha ERD/S (in %) per hemisphere (left, right), group (programmers, non-programmers) and complexity level (low, medium, high) of each task (NIR, FIR, and VDR).

	Programmers			Non-Programmers		
		Left	Right		Left	Right
	*N*	*M (SE)*	*M (SE)*	*N*	*M (SE)*	*M (SE)*
*FIR*						
Low	19	− 36.64 (6.22)^a,b^	− 38.35 (5.55)^d, e^	17	− 56.07 (6.29)	− 51.52 (6.65)
Medium	19	− 52.11 (5.93)^a, c^	− 54.50 (6.04)^d, f^	17	− 61.05 (6.16)	− 56.24 (6.12)
High	19	− 59.75 (3.90)^b, c^	− 58.88 (5.03)^e, f^	17	− 64.56 (4.27)	− 58.62 (5.26)
*NIR*						
Low	18	− 43.99 (4.16)	-46.80 (4.49)	19	− 58.73 (5.25)	− 52.08 (5.21)
Medium	18	− 52.08 (4.41)	− 54.23 (4.16)	19	− 60.69 (4.38)	− 57.01 (4.26)
High	18	− 51.79 (5.01)	− 57.01 (4.04)	19	− 63.34 (4.11)	− 57.51 (4.63)
*VDR*						
Low	20	− 31.35 (7.50)	− 35.21 (7.59)	21	− 39.62 (7.17)	− 32.70 (7.50)
Medium	20	− 45.78 (5.57)	− 46.74 (5.92)	21	− 52.23 (6.29)	− 47.34 (6.57)
High	20	− 41.16 (6.40)	− 40.88 (6.98)	21	− 55.19 (5.02)	− 51.02 (5.37)

*FIR* figural inductive reasoning, *NIR* numerical inductive reasoning, *VDR* verbal deductive reasoning. Superscripted letters indicate significant differences revealed by the post-hoc tests for the interaction effect Complexity*Group.

**Figure 2:**
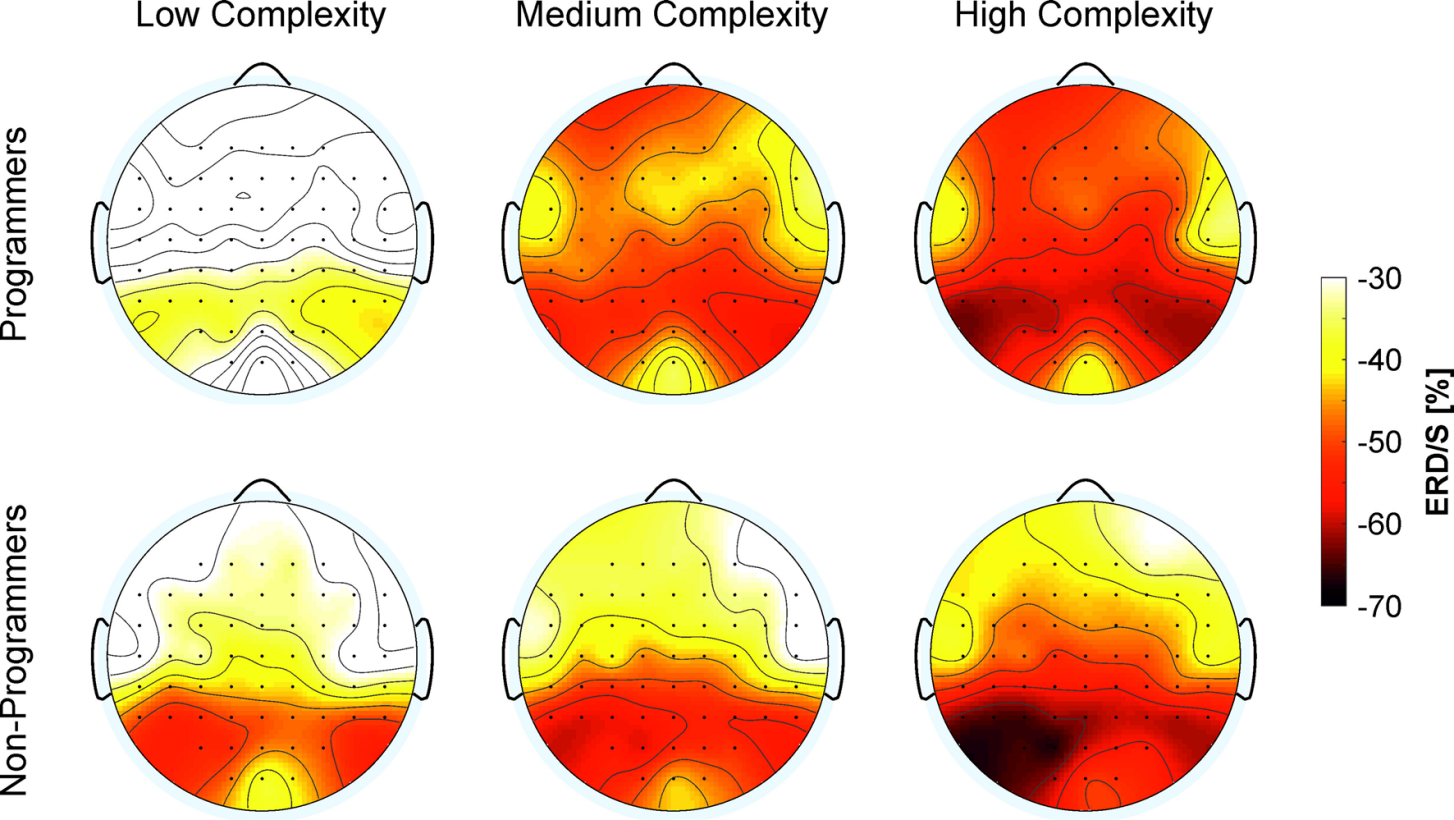
Topographical plots of alpha ERD/S. Topoplots showing alpha ERD/S in programmers and non-programmers in the three complexity levels (low, medium, high) of the Figural Inductive Reasoning task. Only negative values are displayed (ERD). Lower values represent a more pronounced ERD (red), higher values a less pronounced ERD (yellow).

The ANCOVA model for ERD/S values during the FIR task revealed a significant main effect Complexity (*F*(1.66,53.03) = 3.43, *p* < 0.05, *η*_*p*_^*2*^ = 0.10), a significant interaction Complexity*Group (*F*(1.66,53.03) = 5.44, *p* < 0.05, *η*_*p*_^*2*^ = 0.15), and a significant interaction Hemisphere*Group (*F*(1,32) = 6.81, *p* < 0.05, *η*_*p*_^*2*^ = 0.18). Post-hoc comparisons revealed that both programmers and non-programmers showed more pronounced alpha ERD with increasing task complexity (Low vs. Medium: *p* = 0.001; Low vs. High: *p* < αHolm, Medium vs. High: *p* = 0.017; Table 3). In terms of the interaction Complexity*Group, post-hoc comparisons indicated that only programmers showed lower ERD in low complex tasks compared to medium (*p* < 0.001) and highly complex tasks (*p* < 0.001) and lower ERD in medium compared to highly complex tasks (*p* = 0.026) (Table 3, Fig. 2). No such complexity-specific differences were found in non-programmers (Table 3, Fig. 2). Post-hoc comparisons regarding the interaction between Group and Hemisphere found that in non-programmers, alpha ERD was higher in the left than in the right hemisphere (*p* = 0.007). No significant difference between the two hemispheres was found for programmers. Moreover, no differences between programmers and non-programmers were found in any of the two hemispheres (Table 3). The covariates had no significant effects.

For alpha ERD/S during the NIR task, the ANCOVA only revealed a significant interaction of Hemisphere*Group (*F*(1,33) = 13.23, *p* < 0.01, *η*_*p*_^*2*^ = 0.29). Post-hoc comparisons revealed that, within the left hemisphere, non-programmers showed significantly higher ERD than programmers (*p* = 0.039). Additionally, non-programmers showed significantly higher ERD in the left than in the right hemisphere (*p* = 0.003, Table 3).

No significant results were observed for alpha ERD/S in the VDR task (Table 3).

Results of the analysis of theta ERD/S can be found in Supplementary Material C.

### Brain connectivity

Table 4 summarizes the alpha coherence values for each reasoning task and each complexity level per group and hemisphere.

**Table 4:** Means (*M*) and standard errors (*SE*) for fronto-parietal coherence in the alpha frequency range per hemisphere (left, middle, right), group (programmers, non-programmers) and complexity level (low, medium, high) of each task (NIR, FIR, and VDR).

	Programmers				Non-Programmers			
		Left	Middle	Right		Left	Middle	Right
	*N*	*M (SE)*	*M (SE)*	*M (SE)*	*N*	*M (SE)*	*M (SE)*	*M (SE)*
*FIR*								
Low	19	0.038 (0.005)^a^	0.044 (0.009)^b^	0.044 (0.008)^c^	17	0.071 (0.011)^a^	0.073 (0.012)^b^	0.078 (0.012)^c^
Medium	19	0.059 (0.009)	0.074 (0.011)	0.076 (0.009)	17	0.081 (0.011)	0.094 (0.016)	0.092 (0.014)
High	19	0.062 (0.007)	0.077 (0.011)	0.077 (0.012)	17	0.074 (0.011)	0.093 (0.013)	0.087 (0.011)
*NIR*								
Low	19	0.046 (0.010)	0.048 (0.008)	0.047 (0.008)	18	0.057 (0.009)	0.056 (0.010)	0.059 (0.012)
Medium	19	0.048 (0.013)	0.055 (0.012)	0.048 (0.010)	18	0.067 (0.016)	0.067 (0.015)	0.057 (0.015)
High	19	0.066 (0.010)	0.072 (0.012)	0.068 (0.014)	18	0.070 (0.010)	0.080 (0.013)	0.072 (0.011)
*VDR*								
Low	20	0.078 (0.010)	0.079 (0.009)	0.073 (0.009)	21	0.071 (0.008)	0.076 (0.008)	0.067 (0.006)
Medium	20	0.067 (0.007)	0.072 (0.008)	0.072 (0.010)	21	0.086 (0.010)	0.091 (0.008)	0.086 (0.008)
High	20	0.089 (0.014)	0.097 (0.017)	0.087 (0.015)	21	0.129 (0.019)	0.130 (0.019)	0.122 (0.021)

*FIR* figural inductive reasoning, *NIR* numerical inductive reasoning, *VDR* verbal deductive reasoning. Superscripted letters indicate significant differences revealed by the post-hoc tests for the interaction effect Complexity*Group.

For the FIR task, the ANCOVA revealed a significant main effect Complexity (*F*(2,64) = 4.65, *p* < 0.05, *η*_*p*_^*2*^ = 0.12). Post-tests revealed that both programmers and non-programmers showed more pronounced alpha coherence values with increasing task complexity (Low vs. Medium: *p* = 0.002; Low vs. High: *p* = 0.005, Medium vs. High: *ns.*; Table 4). The main effect group was not significant (*F*(1,32) = 2.87, *p* = 0.10, *η*_*p*_^*2*^ = 0.08), however, there was a trend that programmers (*M* = 0.06, *SE* = 0.01) show a lower alpha coherence than non-programmers (*M* = 0.08, *SE* = 0.01). Although the interaction effect Group*Complexity was not significant (*F*(2,64) = 1.31, *p* = 0.26, *η*_*p*_^*2*^ = 0.04), explorative post-t-tests revealed that groups differed significantly in coherence values in the low complexity condition, where non-programmers show higher alpha coherence than programmers (left: *p* = 0.008; middle: *p* = 0.04; right: *p* = 0.02). No group differences were observed in the medium and high complexity condition (Table 4).

In the NIR task, the ANCOVA only revealed a significant main effect of Hemisphere (*F*(1.57, 51.82) = 4.40, *p* < 0.05, *η*_*p*_^*2*^ = 0.12). However, post-hoc tests revealed no significant differences in alpha coherence values between left, middle, and right fronto-parietal connections (Table 4).

The ANCOVA for alpha coherence during the VDR task revealed no significant results (Table 4).

Results of the analysis of theta coherence can be found in Supplementary Material D.

## Discussion

In the present study, we investigated neural processes underlying reasoning (i.e., fluid intelligence) in programmers, who might have developed a form of CT, which is required to program successfully^1,3,10,11,14^, and individuals with no previous programming experience. Programmers showed higher behavioral performance levels as well as a more efficient neural processing in the figural reasoning task compared to non-programmers. No differences in behavior or indices of neural efficiency were observed in the verbal or numerical reasoning tasks. These results are discussed in more detail below.

### Performance differences in figural reasoning tasks

In the figural reasoning task, programmers performed significantly better than non-programmers in the medium and highly complex conditions. No such group differences were observed in the numerical or the verbal reasoning tasks. In the NIR, we observed a general effect of task complexity. All participants, regardless of their group, correctly answered more low than medium or highly complex items and more medium than highly complex items. These findings are compatible with factor-analytical studies showing that individual differences in these three tasks are best explained by a general fluid intelligence factor and modality-specific factors^55–57^.

Our results are in line with previous findings showing that higher programming skills as well as higher CT skills come along with higher figural reasoning skills^14,19,20^. While all three reasoning tests used in the present study require problem-solving abilities, which are highly interrelated with both, programming and CT^3,11,20,79^, FIR specifically requires figural, rather than numerical or verbal processing^14^. Intervention studies in which CT and/or programming skills were trained led to an improvement in figural reasoning tasks but not in numerical or verbal reasoning tasks^13,14,20–23^. For instance, Ambrosio et al.^14^ showed that the grades of college students at the end of their first programming course correlated with their spatial reasoning ability (similarly to FIR task in the present study) at the beginning of the course. Likewise, a meta-analysis on programming interventions discovered a positive influence of the interventions on spatial skills, which include spatial reasoning^13^. Studies, where CT was measured directly (e.g. using a CT test), also found this connection in both adults^20^ and children^23^. Román-González et al.^23^ found a relationship between CT and spatial ability, but no relationship between CT and numerical ability. Boom et al.^20^, detected a high correlation between college students’ CT skills, assessed by items of the Bebras challenge, and FIR, as assessed by a similar test as the one we used in the present study. Román-González et al.^23^ demonstrated that both reasoning and spatial abilities were significant predictors of good performance in their CT test for Spanish school students (grade 5 to 10).

Both FIR and NIR are tests on inductive reasoning and, thus, the difference between the two tests is that only NIR requires numerical processing, which is not assumed to be a part of CT^3^. Therefore, the distinction between CT and numerical abilities^3,14,23^ might explain why programmers outperformed non-programmers in FIR, while performing equally well in NIR. Similarly, verbal processing is important in VDR^80–82^, which does not seem to be highly interrelated with programming and related CT skills^22,23^.

### Differences in mental strategies

The analysis of the mental strategies reported by our participants to solve the reasoning tasks support that, too. When comparing the mental strategies reported by our participants to solve the FIR and the NIR items, it seems as if the NIR items are primarily solved by applying basic mathematical operations, while solving the FIR items required more the use of many different rules (number-, position-, shape-, rotation of objects, etc.), algorithmic thinking (e.g., *if–then* operations), and pattern recognition, which is comparable to mental processes involved in programming^83^. Our results indicate that the ability of figural reasoning is closely related to programming experience and, thus, could be a fundamental component of CT skills, which are required for programming^14,23,84^.

Programmers and non-programmers reported comparable mental strategies to solve the three reasoning tasks. However, the report of a strategy does not reveal anything about its quality or effective usage.

### Differences in brain activity

Only programmers showed differences in brain activity between the three complexity levels of the FIR task. Programmers showed decreases in brain activity with decreases in task complexity. This was indicated by a lower alpha ERD in low complex tasks as compared to medium and highly complex FIR tasks and a lower alpha ERD in medium compared to highly complex FIR tasks. No such complexity-specific differences were found in non-programmers. Hence, the superior behavioral performance in the FIR task in programmers compared to non-programmers goes along with a more efficient allocation of neural processing. Programmers seem to need less neural resources to solve the easier FIR tasks while the non-programmers are already more strongly activated during the easy FIR tasks, although no differences between groups were present in behavioral measurements. Similar results have already been observed in earlier studies^35,85^. Doppelmayr et al.^85^, for instance, compared students regarding their brain activity while working on the RAVEN test, which is similar to FIR in the present study. Based on their performance in the test, students were divided into two groups (higher IQ and lower IQ). While the group with higher IQ showed significantly less upper alpha ERD in easy tasks, no group difference was observed in more difficult tasks. These results were found in several other studies included in a comprehensive review by Neubauer and Fink^35^. Usually, in these studies the results were explained in such a way that better performing individuals are able to increase brain activation with increasing task demands and are willing to invest more effort in complex tasks, being aware that they could solve them^35,85^. Our results are in line with the neural efficiency theory that individuals with higher cognitive skills show a less pronounced or more specific brain activation during task performance^30,31,33,34,45,86^. Neubauer et al.^35,77,87^ also mentioned that neural efficiency has been most consistently found during reasoning and figural-spatial information processing, which might explain why we have only found indicators for neural efficiency in the FIR but not in the NIR or VDR task^35,77,87^.

A more efficient neural processing in programmers might be a sign that programmers showed a stronger involvement of automatic, capacity-free type I cognitive processes, especially in easy FIR tasks, while non-programmers activated more cognitive-demanding type II processes leading to a stronger brain activation. Hence, programmers might have been more likely to process simple patterns and did not need extensive logical reasoning to decide upon the correct answer in easy FIR tasks. The lower brain activation in programmers during easy FIR tasks might be a sign for the use of more efficient brain pathways, which the non-programmers might also develop with increasing programming experience. However, our results support the assumption of a dual-process model of reasoning in which programming experience might lead to a better balance between executive and associative processes^38,39,42^.

An additional discussion of further EEG results can be found in Supplementary Material E.

### Differences in brain connectivity

The results of the connectivity analysis also indicate a more efficient neural processing during figural reasoning in programmers than in non-programmers. Although the interaction effect Group*Complexity was statistically non-significant, we found a trend towards programmers showing lower alpha coherence during easier figural reasoning tasks than non-programmers. This might be a further sign for a higher neural efficiency in programmers than in non-programmers. Programmers seem to need less neural resources as indicated by a reduced fronto-parietal brain connectivity than non-programmers in easier tasks with the same behavioral performance. Our finding of an involvement of a fronto-parietal network in figural reasoning tasks is in line with prior findings^88–90^. Generally, it is assumed that the prefrontal cortex exerts supervisory control over posterior parietal regions^91^. A higher fronto-parietal connectivity in non-programmers when solving reasoning tasks might indicate that frontal areas exerted a stronger supervisory control (type II processes) over parietal areas during this task, while a lower fronto-parietal connectivity in programmers might indicate that posterior systems operated more automatically without the need of frontal executive control in this group, supporting the assumption that programmers show a stronger involvement of type I cognitive control processes during reasoning^41,46,89,91^. In contrast to the present finding, Neubauer and Fink^35^ found a higher functional brain connectivity in higher intelligent individuals than in lower intelligent individuals. However, Neubauer and Fink^35^ used another measure of brain connectivity, namely the phase locking value PVL (magnitude squared coherence). The PVL is a non-linear measure of phase synchronization independent of the signal amplitude. The magnitude-squared coherence is a linear method incorporating phase and amplitude information. Linear and non-linear measures provide different, but complementary information^92^. Therefore, the results of the present study and the study by Neubauer and Fink^35^ are not directly comparable. The results of the connectivity analysis have to be interpreted with caution, since the ANCOVA revealed no significant interaction effect. However, the results of the post-hoc *t*-tests point to a more efficient brain connectivity that is adapting with task complexity in programmers than in non-programmers.

Both groups showed an increase in alpha and theta coherence with increasing task complexity. Hence, a stronger functional fronto-parietal connectivity was observed in more difficult than in less difficult figural reasoning tasks. This indicates that with increasing task complexity, frontal areas need to exert stronger supervisory control over parietal areas^89,91^. Prior studies also showed a lower brain connectivity in easier than in more complex tasks^36,37^.

For theta ERD/S and theta coherence, no meaningful group differences were observed (see Supplementary Material C & D). Prior EEG studies that investigated neural efficiency effects also primarily report on effects in the alpha frequency range and not in theta^32–35,44,45,77,87,93^.

### Limitations and conclusions

We found evidence for stronger neural efficiency probably due to a stronger involvement of automatic, capacity-free type I cognitive control processes in individuals with programming experience than in non-programmers. We assume that programming requires CT skills. Behavioral and neural differences between groups were found only in figural but not in numerical or verbal reasoning tasks. This indicates that programming skills are mainly associated with mental processes involved in figural reasoning but not in numerical reasoning or verbal reasoning. Results of the verbal strategies reported to solve the specific reasoning tasks support that, too.

One limitation of the present study is that we did not assess CT directly using CT tasks. However, due to the lack of a widely accepted definition of CT and the resulting shortage of standardized assessment tools^3,94^, we decided to compare between individuals with and without considerable programming experiences. According to previous literature, programming experience is a strong indicator for CT^3,13,95^, although programming and CT is not equivalent (i.e. CT is assumed to exceed programming)^3,10,11^. We cannot draw any conclusions about neural underpinnings of CT directly based on the present data.

Since we compared participants with and without considerable programming experience, the observed group differences might be attributed to differences in programming experience. Nevertheless, future studies might consider comparing experts and novices, for example, students from higher versus lower semesters of the same study course, or comparing individuals in their behavioral performance and neural processing before and after acquiring programming experience.

Another point is that we did not directly test participants’ programming skills, but assessed the amount of programming experience by self-estimation ratings^13,48,96^. However, there is evidence that programming experience can be reliably assessed using such self-estimation ratings^48^. Additionally, given the high amount of programming experience of the programmer group and the lack of programming experience in non-programmers, it is reasonable to assume that both groups differed considerably concerning their programming skills.

For the analysis of the EEG data, we only included correctly answered items. Hence, especially for the analysis of medium and highly complex FIR items, less trials were included in the EEG analysis for non-programmers than for programmers. This might lead to differences in measurement precision between groups. However, significant differences in alpha ERD were observed within the programmer group across complexity levels, where the difference in the amount of included trials was not so strong. Additionally, it cannot be assumed that items were processed properly if they were not answered correctly. Therefore, we decided to report only on the EEG results of correctly answered items in the present study.

Another limitation of the present study might be the sample size. With the present design, only large effects of *f* > 0.40 can be revealed. However, the present sample size is comparable to the sample size of previous EEG studies that investigated neural efficiency during cognitive tasks reporting on large effects, too (e.g.,^33,47,87,93^).

To conclude, the present study provides further evidence that individuals with programming experience might develop a form of CT, which they can apply on complex problem-solving tasks such as reasoning tests. Since CT is applied in programming, this could provide important information about the concept of CT, which is regarded as a fundamental skill of the twenty-first century^3,13–15^.

## Supplementary information

Supplementary Information.

## Data Availability

Data that support the findings of this study are available on request from the corresponding author (S.E.K.) after contacting the Ethics Committee of the University of Graz (ethikkommission@uni-graz.at) for researchers who meet the criteria for access to confidential data. These ethical restrictions prohibit the authors from making the data set publicly available.
