# Supplementary material for: Programming experience associated with neural efficiency during figural reasoning
Source: Sci Rep. 2020 Aug 7;10:13351. doi: 10.1038/s41598-020-70360-z (PMC7415147; doi:10.1038/s41598-020-70360-z)
Supplement: Supplementary file 1 — Supplementary Information. [file 41598_2020_70360_MOESM1_ESM.docx]

**Supplementary Material**

Title: Programming experience associated with neural efficiency during figural reasoning

Authors: Birgit Helmlinger^1^, Markus Sommer^1^, Martina Feldhammer-Kahr^1^, Guilherme Wood^1, 2^, Martin E. Arendasy^1^, *Silvia E. Kober^1, 2^

1- Institute of Psychology, University of Graz, Graz, Austria

2- BioTechMed-Graz, Graz, Austria

*Corresponding author:

Silvia Erika Kober

Institute of Psychology, University of Graz

Universitätsplatz 2, 8010, Graz, Austria

Tel. +43 316 380 8497

E-Mail: silvia.kober@uni-graz.at

**Supplementary Material A** - Programming experience of programmers and non-programmers

Table A1

*Information about programming experience and prior education of each participant of the programmer group (participant number 1-20) and the non-programmer group (participant number 21-41)*

| Partici-pant Nr. | Sex | Age | Courses of Study | Degree completed | Programm-ing courses at school | Number of semesters in which programm-ing courses were taught at school | Number of finished programm-ing courses during study | Programming language | Programm-ing during leisure time | Expertise rating |
| --- | --- | --- | --- | --- | --- | --- | --- | --- | --- | --- |
| 1 | M | 21 | Computer Science | N | Y | 8 | 13 | Java, HTML, C, C++, Verilog, Assembler, Python, Matlab, PHP | Y | 8 |
| 2 | M | 22 | Biomedical Engineering | N | Y | 8 | 2 | C#, Assembler, C | Y | 5 |
| 3 | M | 23 | Software Engineering and Management | N | Y | 6 | 5 | C, C++, Java, Python | N | 8 |
| 4 | M | 23 | - | N | Y | 4 | - | C++, C#, SPS (FUP), SQL | Y | 6 |
| 5 | M | 23 | Mathematics | Y | N | - | 3 | Diverse | Y | 5 |
| 6 | M | 24 | Electrical Engineering | Y | Y | 4 | 2 | C, C#, SCL | Y | 8 |
| 7 | M | 24 | Electrical Engineering | N | Y | 8 | 2 | Assembler, C, Java, Matlab, SPS (AWL, KOP, FUP, ANSI C) | Y | 7 |
| 8 | M | 25 | Production Technology and Organization | N | Y | 4 | 10 | C#, HTML, PHP, C++, Java | Y | 8 |
| 9 | M | 25 | Software Engineering and Management | Y | N | - | 20 | Diverse | Y | 6 |
| 10 | M | 26 | Electrical Engineering | N | Y | 4 | 5 | C# | N | 6 |
| 11 | M | 29 | Computer Science | Y | Y | 10 | 30 | Java, PHP, JavaScript, Actionscript | Y | 10 |
| 12 | M | 29 | Environmental Systems Sciences | Y | Y | 8 | 3 | Pascal, C | Y | 6 |
| 13 | F | 19 | Environmental Systems Sciences | N | Y | 6 | 1 | C, C++, Java, HTML | Y | 5 |
| 14 | F | 23 | Biomedical Engineering | N | N |  | 4 | Diverse | N | 7 |
| 15 | F | 23 | Computer Science | Y | Y | 8 | 25 | Java, C | N | 7 |
| 16 | F | 26 | Environmental Systems Sciences | N | N | - | 5 | Java, SQL | Y | 6 |
| 17 | F | 26 | Psychology | Y | N | - | - | Python, Ruby on Rails apps, Java, HTML/CSS | Y | 6 |
| 18 | F | 26 | Psychology | Y | Y | 2 | 3 | HTML, Matlab, R, Python | N | 5 |
| 19 | F | 28 | Physics | Y | N | - | 5 | Diverse | N | 7 |
| 20 | F | 38 | Psychology | Y | N | - | - | Java, C# | N | 7 |
| 21 | M | 19 | Teaching Degree Mathematics/English | N | N | - | - | - | N | 0 |
| 22 | M | 21 | Musicology | N | N | - | - | - | N | 0 |
| 23 | M | 22 | Psychology | N | N | - | - | - | N | 0 |
| 24 | M | 24 | Psychology | N | N | - | - | - | N | 0 |
| 25 | M | 25 | Law | N | N | - | - | - | N | 0 |
| 26 | M | 25 | Psychology | N | N | - | - | - | N | 0 |
| 27 | M | 26 | Psychology | N | N | - | - | - | N | 0 |
| 28 | M | 26 | Psychology | N | N | - | - | - | N | 0 |
| 29 | M | 27 | Global Studies | Y | N | - | - | - | N | 0 |
| 30 | M | 27 | Musicology | N | N | - | - | - | N | 0 |
| 31 | F | 19 | Pharmaceutical Sciences | N | N | - | - | - | N | 0 |
| 32 | F | 19 | Molecular Biology | N | N | - | - | - | N | 0 |
| 33 | F | 19 | Molecular Biology | N | N | - | - | - | N | 0 |
| 34 | F | 21 | Molecular Biology | N | N | - | - | - | N | 0 |
| 35 | F | 23 | Psychology | N | N | - | - | - | N | 0 |
| 36 | F | 23 | Law | N | N | - | - | - | N | 0 |
| 37 | F | 23 | Applied Ethics | Y | N | - | - | - | N | 0 |
| 38 | F | 24 | Translation | Y | N | - | - | - | N | 0 |
| 39 | F | 28 | Psychology | Y | N | - | - | - | N | 0 |
| 40 | F | 30 | Molecular Biology | N | N | - | - | - | N | 0 |
| 41 | F | 35 | Social Pedagogy | Y | N | - | - | - | N | 0 |

*Note*. F = female, M = male, N = no, Y = yes.

**Supplementary Material B** - Analysis and results of EEG resting measurements with open and closed eyes before reasoning tasks

EEG resting measurements with open and closed eyes were performed before the reasoning tasks to investigate whether groups are comparable in their resting EEG. For instance, possible differences in EEG resting power might restrict comparability of ERD/ERS values (task-related power changes between baseline and active phase during reasoning tasks).

The EEG resting measurements with open and closed eyes were preprocessed in the same way as the EEG data assessed during the reasoning tasks (see section *2.3 EEG recording and data analysis* of the main manuscript). Alpha- (8 – 12 Hz) and theta band power (4 – 8 Hz) were extracted and averaged over artifact free epochs of the whole one-minute resting measurements, separately for the eyes open and eyes closed condition. Average artifact-free EEG coherence values were also extracted for the frequency range of 4-8 Hz and 8-12 Hz per condition (eyes open/closed).

To analyze the resting EEG data, an ANCOVA with the between-subjects-factor Group (programmers, non-programmers) and the within-subjects factor ROIs (left, right) was calculated for the dependent variable alpha power and an ANCOVA with the between-subjects-factor Group (programmers, non-programmers) for the dependent variable theta power, separately for the eyes open and eyes closed condition. Additionally, ANCOVAs with the between-subjects-factor Group (programmers, non-programmers) and the within-subjects factor Hemisphere (left, middle, and right fronto-parietal connections) were calculated for the dependent variable alpha and theta coherence, separately for the eyes open and eyes closed condition. Age and sex of the participants were used as covariates.

The ANCOVAs for the dependent variable EEG alpha and theta power during the resting conditions with open and closed eyes revealed no significant effects. Groups were comparable in resting EEG alpha and theta power. Hence, groups only differed in task-related alpha power changes (see *Results* section of the main manuscript) but not in the baseline alpha or theta power, as assessed during resting measurements with open and closed eyes. This indicates that the group differences in task-related alpha power changes during the FIR task cannot be caused by intrinsic group differences in alpha power during rest.

The ANCOVAs for alpha and theta coherence during the eyes open resting condition revealed no significant effects.

The ANCOVA for alpha coherence during the eyes closed resting condition revealed a significant main effect Group (*F*(1,34) = 8.11, *p* < 0.01, *η_p_²* = 0.19). Programmers (*M* = 0.085, *SE* = 0.009) showed a higher alpha coherence during this resting condition than non-programmers (*M* = 0.049, *SE* = 0.009).

The ANCOVA for theta coherence during the eyes closed resting condition revealed no significant effects.

The results of the resting measurements support previous studies, showing a higher fronto-parietal brain connectivity in people with higher computational thinking (CT) skills, programming skills, or intelligence during resting conditions [1–4]. For instance, Walden et al. (2015) could show that learning to program led to increased connectivity between frontal and parietal brain regions as well as within prefrontal areas [4].

**Supplementary Material C** – Results of task-specific EEG theta power changes (Theta ERD/S)

Table C1 summarizes the theta ERD/S values for each reasoning task and each complexity level per group.

Table C1

*Means (M) and standard errors (SE) for frontal theta ERD/S (in %) in each reasoning task (FIR, NIR, VDR), presented separately for each complexity level (low, medium, high) and group (programmers, non-programmers)*

|  |  | Programmers | | |  | Non-Programmers | | |
| --- | --- | --- | --- | --- | --- | --- | --- | --- |
|  |  | *N* | *M* | *SE* |  | *N* | *M* | *SE* |
| FIR | low | 20 | 17.39 | 4.83 |  | 16 | 9.28 | 8.14 |
|  | medium | 20 | 21.47 | 5.79 |  | 16 | 6.18 | 8.74 |
|  | high | 20 | 22.78 | 7.31 |  | 16 | 6.00 | 8.58 |
| NIR | low | 17 | 10.59 | 5.23 |  | 19 | 16.78 | 7.40 |
|  | medium | 17 | 14.90 | 5.38 |  | 19 | 10.92 | 7.63 |
|  | high | 17 | 25.46 | 7.06 |  | 19 | 23.94 | 10.74 |
| VDR | low | 19 | 6.33 | 4.94 |  | 21 | -9.07 | 5.87 |
|  | medium | 19 | -3.24^a^ | 3.87 |  | 21 | -2.37 | 5.97 |
|  | high | 19 | 6.16^a^ | 3.13 |  | 21 | -3.84 | 5.58 |

*Note*. NP = Non-Programmers, P = Programmers, FIR = Figural Inductive Reasoning, NIR = Numerical Inductive Reasoning, VDR = Verbal Deductive Reasoning. Superscripted letters indicate significant differences revealed by the post-hoc tests for the interaction effect Complexity*Group.

The ANCOVA models revealed neither significant main effects, nor significant interaction effects regarding frontal theta ERD/S in the FIR and NIR task. In the VDR task, there was a significant interaction Complexity*Group (*F*(2,72) = 3.28, *p* < 0.05, *η_p_²* = 0.08). Post-hoc comparisons revealed that programmers showed significantly lower ERD/S-values (a slight ERD) in medium compared to highly complex tasks (a slight ERS, *p* = .015, Table C1). No other comparisons reached significance.

**Supplementary Material D** – Results of analysis regarding theta coherence

Table D1 summarizes the theta coherence values for each reasoning task and each complexity level per group and hemisphere.

Table D1

*Means (M) and standard errors (SE) for fronto-parietal coherence in the theta frequency range per hemisphere (left, middle, right), group (programmers, non-programmers) and complexity level (low, medium, high) of each test (NIR, FIR and VDR)*

|  |  | Programmers | | | |  | Non-Programmers | | | |  |
| --- | --- | --- | --- | --- | --- | --- | --- | --- | --- | --- | --- |
|  |  |  | left | middle | right |  |  | left | middle | right | |
|  |  | *N* | *M (SE)* | *M (SE)* | *M (SE)* |  | *N* | *M (SE)* | *M (SE)* | *M (SE)* | |
| FIR | low | 19 | 0.027(0.003) | 0.030(0.003) | 0.033(0.005) |  | 17 | 0.029(0.004) | 0.032(0.004) | 0.032(0.004) | |
|  | medium | 19 | 0.041(0.004) | 0.043(0.004) | 0.048(0.006) |  | 17 | 0.047(0.006) | 0.056(0.008) | 0.060(0.007) | |
|  | high | 19 | 0.053(0.006) | 0.059(0.007) | 0.062(0.009) |  | 17 | 0.057(0.007) | 0.063(0.008) | 0.060(0.005) | |
| NIR | low | 19 | 0.030(0.004) | 0.032(0.005) | 0.034(0.006) |  | 18 | 0.039(0.008) | 0.041(0.010) | 0.039(0.008) | |
|  | medium | 19 | 0.035(0.007) | 0.035(0.006) | 0.035(0.005) |  | 18 | 0.041(0.008) | 0.044(0.009) | 0.040(0.007) | |
|  | high | 19 | 0.054(0.009) | 0.050(0.009) | 0.050(0.010) |  | 18 | 0.050(0.009) | 0.056(0.009) | 0.054(0.007) | |
| VDR | low | 20 | 0.081(0.015) | 0.080(0.012) | 0.079(0.013) |  | 21 | 0.069(0.008) | 0.077(0.008) | 0.067(0.006) | |
|  | medium | 20 | 0.061(0.011) | 0.061(0.011) | 0.061(0.010) |  | 21 | 0.072(0.010) | 0.076(0.012) | 0.069(0.010) | |
|  | high | 20 | 0.079(0.011) | 0.078(0.012) | 0.092(0.016) |  | 21 | 0.088(0.015) | 0.099(0.015) | 0.096(0.014) | |

*Note*. NP = Non-Programmers, P = Programmers, FIR = Figural Inductive Reasoning, NIR = Numerical Inductive Reasoning, VDR = Verbal Deductive Reasoning

In the FIR task, the ANCOVA revealed a significant main effect Complexity (*F*(2,66) = 10.02, *p* < 0.001, *η_p_²* = 0.23). Post tests revealed that both programmers and non-programmers showed more pronounced theta coherence values with increasing task complexity (Low vs. Medium: *p* < .0001; Low vs. High: *p* < .0001, Medium vs. High: ns.; Table D1). The main effect Hemisphere was significant, too (*F*(2,66) = 5.16, *p* < 0.01, *η_p_²* = 0.14). Post-tests showed that theta coherence was larger over the right than over the left hemisphere (*p* = 0.006). Moreover, theta coherence was larger over central areas than over the left hemisphere (*p* = 0.011).

No significant effects were observed for theta coherence during the NIR and VDR task (Table D1).

**Supplementary Material E** – Additional discussion

Groups only differed in task-related alpha power changes but not in the baseline alpha or theta power, as assessed during resting measurements with open and closed eyes. This indicates that the group differences in task-related alpha power changes during the FIR task cannot be caused by intrinsic group differences in alpha power during rest.

Non-Programmers exhibited a significantly higher activation in the left than in the right hemisphere according to the alpha ERD of the FIR task. Programmers showed no hemisphere differences in brain activation. According to prior studies, the right hemisphere seems to be important for spatial processing in such spatial reasoning tasks [5, 6]. In the present study, non-programmers exhibited less activation in the right parieto-occipital areas and, additionally, performed inferiorly than programmers. Thus, this might be a further sign of a less efficient neural processing in non-programmers during the FIR task.

Similarly to FIR, non-programmers also displayed lower left-hemispheric activation (alpha ERD) compared to the right hemisphere in NIR. No such hemispheric difference was observed in programmers. Higher left-hemispheric activation of parietal regions has been associated with retrieving important information, for example intermediate results, or verbal number processing within easier tasks [7, 8]. However, within complex problems, activation gets more pronounced and becomes rather bilateral [7, 8]. This bilateral activation enables to mentally represent and manipulate numerical quantities and, thus, permits procedural processing of arithmetic problems [9–13]. Programmers showed this bilateral activation and, additionally, exhibited less activation than non-programmers in the left hemisphere, but performed equally well in the test. Therefore, this result indicates that, although there was no significant difference in performance between programmers and non-programmers in the NIR task, programmers processed the tasks more efficiently.

Concerning alpha ERD/S in VDR, which is a test where processing of verbal information is required, no significant differences between programmers and non-programmers were observed. According to a recent meta-analysis, the inferior parietal cortex is equally involved in spatial and verbal reasoning processes [14]. This might contribute to the assumption formed by behavioral analyses, that programmers did only show more efficiency in tasks with higher proximity to programming.

References

1. Lee, T.-W., Wu, Y.-T., Yu, Y.W.-Y., Wu, H.-C., Chen, T.-J.: A smarter brain is associated with stronger neural interaction in healthy young females: A resting EEG coherence study. Intelligence **40**(1), 38–48 (2012). doi: 10.1016/j.intell.2011.11.001

2. Jung, R.E., Haier, R.J.: The Parieto-Frontal Integration Theory (P-FIT) of intelligence: converging neuroimaging evidence. The Behavioral and brain sciences **30**(2), 135-54; discussion 154-87 (2007). doi: 10.1017/S0140525X07001185

3. Song, M., Zhou, Y., Li, J., Liu, Y., Tian, L., Yu, C., Jiang, T.: Brain spontaneous functional connectivity and intelligence. NeuroImage **41**(3), 1168–1176 (2008). doi: 10.1016/j.neuroimage.2008.02.036

4. Walden, E., Browne, G., Oboyle, M.: Computational Thinking: Changes to the Human Connectome Associated with Learning to Program. Thirty Sixth International Conference on Information Systems, Fort Worth, 1–14 (2015)

5. Dix, A., Wartenburger, I., van der Meer, E.: The role of fluid intelligence and learning in analogical reasoning: How to become neurally efficient? Neurobiology of learning and memory **134 Pt B**, 236–247 (2016). doi: 10.1016/j.nlm.2016.07.019

6. Vogel, J.J., Bowers, C.A., Vogel, D.S.: Cerebral lateralization of spatial abilities: A meta-analysis. Brain and cognition **52**(2), 197–204 (2003). doi: 10.1016/S0278-2626(03)00056-3

7. Wood, G., Nuerk, H.-C., Moeller, K., Geppert, B., Schnitker, R., Weber, J., Willmes, K.: All for one but not one for all: how multiple number representations are recruited in one numerical task. Brain research **1187**, 154–166 (2008). doi: 10.1016/j.brainres.2007.09.094

8. Grabner, R.H., Ansari, D., Reishofer, G., Stern, E., Ebner, F., Neuper, C.: Individual differences in mathematical competence predict parietal brain activation during mental calculation. NeuroImage **38**(2), 346–356 (2007). doi: 10.1016/j.neuroimage.2007.07.041

9. Dehaene, S., Cohen, L.: Cerebral Pathways for Calculation: Double Dissociation between Rote Verbal and Quantitative Knowledge of Arithmetic. Cortex **33**(2), 219–250 (1997). doi: 10.1016/S0010-9452(08)70002-9

10. Dehaene, S., Molko, N., Cohen, L., Wilson, A.J.: Arithmetic and the brain. Current Opinion in Neurobiology **14**(2), 218–224 (2004). doi: 10.1016/j.conb.2004.03.008

11. Grabner, R.H., Smedt, B. de: Neurophysiological evidence for the validity of verbal strategy reports in mental arithmetic. Biological psychology **87**(1), 128–136 (2011). doi: 10.1016/j.biopsycho.2011.02.019

12. Tschentscher, N., Hauk, O.: How are things adding up? Neural differences between arithmetic operations are due to general problem solving strategies. NeuroImage **92**, 369–380 (2014). doi: 10.1016/j.neuroimage.2014.01.061

13. Sakkalis, V., Zervakis, M., Micheloyannis, S.: Significant EEG Features Involved in Mathematical Reasoning: Evidence from Wavelet Analysis. Brain Topogr **19**(1), 53–60 (2006). doi: 10.1007/s10548-006-0012-z

14. Santarnecchi, E., Khanna, A.R., Musaeus, C.S., Benwell, C.S.Y., Davila, P., Farzan, F., Matham, S., Pascual-Leone, A., Shafi, M.M.: EEG Microstate Correlates of Fluid Intelligence and Response to Cognitive Training. Brain topography **30**(4), 502–520 (2017). doi: 10.1007/s10548-017-0565-z
